# Peptide-substituted oligonucleotide synthesis and non-toxic, passive cell delivery

**DOI:** 10.1038/sigtrans.2016.19

**Published:** 2016-10-21

**Authors:** Shiying Shang, Luca Monfregola, Marvin H Caruthers

**Affiliations:** 1 Department of Chemistry and Biochemistry, University of Colorado, Boulder, Colorado, USA

## Abstract

Chemically modified oligodeoxynucleotides (ODNs) are known to modulate gene expression by interacting with RNA. An efficient approach for synthesizing amino acid- or peptide-substituted triazolylphosphonate analogs (TP ODNs) has been developed to provide improved stability and cell uptake. The chemistry is quite general, as peptides can be introduced throughout the TP ODN at any preselected internucleotide linkage. These synthetic TP ODNs enter cells through endocytosis in the absence of transfection reagents and localize into perinuclear organelles. The entrapped ODNs are released into the cytoplasm by treatment with endosomal-releasing agents and several are then active as microRNA inhibitors.

## Introduction

Oligodeoxynucleotides (ODNs) are a class of therapeutic agents that can be used for antisense, RNA interference, immunorecognition and aptamer binding.^1–3^ Although many ODN analogs have been developed for biological applications, none are completely satisfactory and further improvements in certain properties are required before the full therapeutic value of these molecules can be realized. Properties to be considered are stability, cellular uptake efficiency, site-specific delivery and off-target effects.

Triazolylphosphonate DNA analogs can be synthesized efficiently on a solid support and undergo endocytosis by multiple cell types *in vitro* in the absence of transfection lipids.^4^ To further neutralize anionic DNA and therefore improve the cellular uptake of these analogs, we have extended this modification to more positively charged, peptide-substituted 1,2,3-triazolyl-phosphonate DNA analogs (TP ODNs). Notably, the precise charge and location of these modifications can be fine-tuned by structural variations.

Peptides can often improve the biological potency of oligonucleotides, such as cell-specific delivery, cellular uptake efficiency, intracellular distribution and target specificity. Thus, development of efficient and reproducible methods for the convenient preparation of covalently linked peptide–oligonucleotide conjugates (POCs) has become a subject of considerable importance.^5,6^

Although peptide and oligonucleotide synthesis strategies have been extensively developed, preparing a POC is less straightforward because their chemistries are not fully compatible. Therefore, the synthesis of POCs fall into two general categories: (1) the stepwise, solid-phase synthesis approach where the POC is prepared on a single support using protecting groups that are compatible with amino acids and nucleosides as well as modified protocols that minimize side reactions. (2) The post-synthetic conjugation approach where the oligonucleotide and peptide are prepared using standard chemistries but are functionalized with various linkers so they can be joined post-synthetically. However, both of these approaches are far from routine because, for each route, there are advantages and disadvantages. For the post-synthetic approach, conjugation yields are modest at best, whereas with the stepwise approach, the incompatibility of deprotection and assembly chemistries is a major issue. In addition and because of limitations with these chemistries, the synthesis of POCs by either approach has focused primarily on joining peptides to either the 3′ or 5′ ends of oligonucleotides.^7–9^

In this contribution, we outline a procedure for synthesizing TP ODNs having lysine or peptide-substituted 1,2,3-triazolylphosphonate internucleotide linkages. By choosing this linkage, we present a versatile approach for joining peptides and oligonucleotides at any preselected position. A comparable approach has recently been developed where 2′-alkynyl-2′-amino-LNA in combination with click chemistry is used for peptide insertion at any predefined site in an oligonucleotide.^10^

To explore biological activity, we focused our attention on a non-toxic oligonucleotide delivery method known as ‘passive transfection’.^11^ This procedure does not require any special vehicles such as liposomes, electroporation or microinjection for entry of the oligonucleotide into cells and for it to be biologically active. Using flow cytometry and microscopy, we observed that HeLa cells could efficiently take up these TP ODNs in the absence of lipid transfection reagents. We have also investigated the intracellular localization and anti-microRNA inhibiting ability of TP ODNs targeted to miR-15b. Confocal microscopy showed that the intracellular distribution of these passively transfected TP ODNs was predominantly located in the perinuclear region of HeLa cells. The miR-15b inhibiting ability showed significant activities following lipid transfection and moderate activities from passive transfection. In addition, the passive transfection efficacy was improved through endosomal release.

## Materials and methods

### Azido peptide synthesis

Azido peptides were assembled on a Rink-amide resin to increase resistance towards degradation and also to provide an uncharged C-terminal amide. The synthesized peptides contained an azido group either at the N terminus or on an amino-acid side chain. Peptides were prepared from commercially available Fmoc-Lys(Boc)-OH, Fmoc-Arg(Pbf)-OH, Fmoc-His(^*t*^Bu)-OH, Fmoc-Gly-OH, Fmoc-Lys(N_3_)-OH and N_3_-Gly-OH using the standard Fmoc/HBTU protocol. Acetylation before cleavage generated N-terminal acetylated peptides. After synthesis, peptides were deprotected and cleaved from supports using 95/2.5/2.5 trifluoroacetic acid/triisopropylsilane/water at room temperature and the resins were removed via filtration. Following precipitation in ether, the reaction mixtures were dissolved in an acetonitrile/water solution and pure peptides isolated by high-performance liquid chromatography.

### Alkynyl ODN synthesis

5′-*O*-Dimethoxytrityl-2′-deoxyribonucleoside 3′-*O*-ethynylphosphinoamidite synthons (alkynylphosphinoamidite synthons) were used to synthesize alkynyl linked oligonucleotides using the standard DNA solid-phase synthesis cycle with ETT as activator (5-ethylthio-1H-tetrazole, 0.25 M in anhydrous acetonitrile) and an extended reaction time of 600 s per cycle. 5′-*O*-Dimethoxytrityl-2′-deoxyribonucleoside 3′-*O*-cyanoethylphosphoramidite synthons were used to introduce phosphate internucleotide linkages into alkynyl DNA using the standard 25 s per cycle reaction time. Preparation of the alkynylphosphinoamidite synthons has been described previously.^4^

### Click chemistry

Following the synthesis of a 2′-deoxyoligonucleotide having internucleotide alkynylphosphonates,^4^ these linkages were functionalized, using click chemistry, with azide-containing peptides or amino acids to form peptide or amino-acid-substituted triazolylphosphonate linkages. A heterogeneous mixture of the alkynylphosphonate oligonucleotide joined to the support (1 equiv., 1.0 μM), an azide-containing peptide or amino acid (6 equiv.) in H_2_O/MeOH/THF (1.2 ml, 2:2:1 v/v/v), CuSO_4_·5H_2_O (0.8 equiv.), freshly prepared sodium ascorbate (6 equiv.) and tris[(1-benzyl-1H-1,2,3-triazol-4-yl)methyl]amine (7 equiv.), was allowed to react overnight at room temperature to generate the TP ODN. The resin was then washed successively with water, methanol and dichloromethane (2×10 ml). After drying, cleavage of the reaction mixture from the support was carried out over 2 h at room temperature in a 1:1 solution of anhydrous ethylenediamine in toluene. Solvents were removed next. The reaction mixture containing the product TP ODN was then eluted from the support with water and the TP ODN isolated by purification on high-performance liquid chromatography. A broad product peak was observed due to the presence of multiple chiral phosphorus centers. If needed for various experiments, a fluorescein tag was added to the TP ODN (FL TP ODN) as the last synthesis step before carrying out the click chemistry.

### Melting temperature measurements

Melting temperature (*T*_m_) measurements were performed on a ultraviolet–visible spectrophotometer equipped with a thermoelectrically controlled multicell holder. Equimolar concentrations of TP ODNs and complementary, unmodified DNAs were prepared in a buffer containing 100 mM NaCl and 100 mM sodium phosphate (pH 7.2). The solutions were heated to 90 °C for 5 min followed by cooling to 15 °C at a rate of 1 °C min^−1^, equilibrated for 5 min and then heated to 90 °C at the same rate. Absorbances at 260 nm were recorded throughout at intervals of 0.5 °C. The derivatives of the heating curves were calculated and temperatures corresponding to the maximum of the derivative curves were determined to be the melting temperatures.

### HeLa cells

HeLa cells were purchased from American Type Culture Collection (Manassas, VA, USA) and maintained as monolayer cultures in Dulbecco’s modified Eagle medium (DMEM) containing 10% fetal bovine serum (FBS), penicillin and streptomycin (Pen–Strep, 100 U ml^−1^). Cells were grown in a humidified atmosphere of 5% carbon dioxide at 37 °C. They were counted and adjusted to the appropriate density, seeded onto plates or chambers, and incubated for 24 h before transfection.

### 15b HeLa cells

15b HeLa cells were obtained from miRagen Therapeutics, Inc. (Boulder, CO, USA) and maintained as monolayer cultures in DMEM medium containing 10% FBS, 100 U ml^−1^ Pen–Strep, 1× GlutaMAX, 1× non-essential amino acids, 1× sodium pyruvate and 1× puromycin. Cells were grown in a humidified atmosphere of 5% carbon dioxide at 37 °C. They were counted and adjusted to the appropriate density, seeded onto plates or chambers, and incubated for 24 h before transfection.

### FACS analysis

HeLa cells were seeded at 8×10^4^ cells per well in 12-well plates in DMEM medium containing 10% FBS and Pen–Strep (100 U ml^−1^). The concentration of a 5′-fluorescein-labeled TP ODN in HyPure Molecular Biology Grade Water (Thermo Scientific, Chino, CA, USA) was measured by ultraviolet spectroscopy. After 24 h, the medium was removed and the cells were transfected with the appropriate TP ODN in DMEM to give the required final concentration. The cells were then incubated at 37 °C for 16 h. After incubation, the medium was removed from wells and the cells were washed three times with Dulbecco’s phosphate-buffered saline (D-PBS). Cells were then treated for 3 min at 37 °C with a pre-warmed solution of trypsin–EDTA (1×) until all cells became round and detached from the plates. The cells from each plate were placed in 1 ml D-PBS and pelleted by centrifugation at 1000 r.p.m. for 5 min. The pellets were washed and resuspended in D-PBS, and kept at 0 °C in the dark until analyzed by flow cytometry.

Flow cytometric data on at least 10 000 cells per sample were acquired on a Moflow flow-cytometer (Beckman Coulter, Brea, CA, USA) equipped with a single 488 nm argon laser and a 530/40 nm emission filter (fluorescein). Raw flow cytometry data were manipulated and visualized using Summit 4.3 software (Beckman Coulter). Fluorescence intensity of the 5′-fluorescein tag was analyzed for cells presenting higher fluorescence than the background. The background was defined as the autofluorescence of cells.

### Lipid transfection as observed by microscope imaging

A TP ODN stock solution was diluted with 100 μl OptiMEM (Invitrogen, Carlsbad, CA, USA) to a final concentration of 0.1 μM TP ODN. In a separate eppendorf tube, 2.5 μl DharmaFECT 1 (Dharmacon, Lafayette, CO, USA) was diluted with 100 μl OptiMEM (Invitrogen). The 100 μl solution of the TP ODN and DhamaFECT 1 (Dharmacon) solution were mixed, allowed to equilibrate for 20 min, and 300 μl OptiMEM (Invitrogen) was added. Hela cells were seeded at 0.1×10^6^ cells per well in four-well chambered coverslides (Thermo Scientific Nunc Lab-Tek II Chambered Coverglass) in DMEM medium containing 10% FBS. After 24 h, medium was removed and the cells were washed twice (0.5 ml D-PBS per wash) before transfection at 80% confluency. D-PBS was removed from the HeLa cells, and 250 μl of the transfection mixture was added to each well. Cells were then incubated at 37 °C for 18 h, washed twice (0.5 ml D-PBS per wash) and analyzed in OptiMEM (Invitrogen) using a confocal microscope.

### Passive transfection as observed by microscope imaging

Hela cells were seeded in DMEM medium containing 10% FBS at 0.1×10^6^ cells per well in four-well chambered coverslides (Thermo Scientific Nunc Lab-Tek II Chambered Coverglass). After 24 h, the medium was removed and the cells were washed twice (0.5 ml D-PBS/wash) before transfection at 80% confluency. A TP ODN stock solution was diluted to the desired concentration in OptiMEM (Invitrogen) and this transfection solution (250 μl) was added to each well. Cells were then incubated at 37 °C for the desired time (15–90 h), washed twice (0.5 ml D-PBS/wash) and analyzed in OptiMEM (Invitrogen) using a confocal microscope.

### Colocalization imaging

The Gal-T marker was a gift from Professor Amy Palmer, Department of Chemistry and Biochemistry, University of Colorado at Boulder, Boulder, CO, USA. For colocalization experiments, 0.5 μl Gal-T (0.5 μg μl^−1^) marker was mixed with 1.25 μl TransIT-LT1 (Mirus Bio, Madison, WI, USA) in 62.5 μl OptiMEM (Invitrogen). After incubation at room temperature for 30 min, this complex was diluted with 0.5 ml OptiMEM (Invitrogen), and then 250 μl of the mixture was added to HeLa cells in each well. Cells were incubated at 37 °C for 24 h before passive transfection.

### Cell synchronization

Cells were synchronized through a double thymidine block. At 25–30% confluency, HeLa cells were washed twice with D-PBS, a solution of 2 mM thymidine in complete medium (DMEM with 10% FBS, 100 U ml^−1^ Pen–Strep, 1× GlutaMAX, 1× non-essential amino acids, 1× sodium pyruvate and 1× puromycin) was added, and the cells were incubated for 18 h. After the first block, thymidine was removed. The cells were washed with D-PBS, fresh medium was added and the cells were incubated for 9 h to release the block. Cells were then washed with D-PBS and added to 2 mM thymidine in complete medium for 17 h. After the second block, thymidine was removed. The cells were washed with D-PBS, trypsinized and re-plated in an eight-well chamber with fresh, complete medium. After 7 h incubation to attain cell attachment, the medium was removed and replaced with a solution of 300 μl OptiMEM (Invitrogen) containing 10% FBS and 1 μM TP ODN. Cell division was monitored continuously for 26 h by confocal microscopy.

### Lipid transfection in a dual-luciferase assay

To 50 μl of a reduced serum, no antibiotic medium (2% FBS, 1× GlutaMAX, 1× non-essential amino acids and 1× sodium pyruvate) was added a stock solution of a TP ODN in water (Hyclone Cell Molecular biology Grade, Invitrogen) so as to obtain a final concentration of 0.1 μM TP ODN. In an eppendorf tube, 0.25 μl DharmaFECT 1 (purchased from Dharmacon/ThermoFisher, now GE Dharmacon, Lafayette, CO, USA) was added to 50 μl of the reduced serum medium and this solution was added to the TP ODN in the same medium. TP ODN and DharmaFECT 1 (Dharmacon) were mixed and allowed to form a complex at room temperature (20 min). A measure of 275 μl medium was added to the above complex. Medium was removed from 15b HeLa cells in a 96-well plate and 100 μl of the transfection mixture was added to each well. After 24 h of lipid transfection, cells were collected for luciferase assays.

### Passive transfection as in a dual-luciferase assay

A TP ODN stock solution in water (Hyclone Cell Molecular biology Grade, Invitrogen) was added to 375 μl reduced serum without antibiotic medium (2% FBS, 1× GlutaMAX, 1× non-essential amino acids and 1× sodium pyruvate) so as to obtain a final concentration of 1 μM TP ODN. Medium was removed from 15b HeLa cells in a 96-well plate and 100 μl of the transfection solution was added to each well. After 72 h of passive transfection, cells were collected for luciferase assays.

### Dual-luciferase assays

The stably transfected 15b cell line was a gift from miRagen Therapeutics, Inc. Cells were seeded in DMEM medium containing 10% FBS at 5000 cells per well in 96-well plates. Cells were then incubated at 37 °C for 24 h before transfection. For endosomal release, cells were pretreated with chloroquine (CQ; 100 μM, 100 μl per well) for 3 h in reduced serum medium without antibiotics (DMEM with 2% FBS, 1× GlutaMAX, 1× non-essential amino acids and 1× sodium pyruvate) before lipid (0.1 μM ODN, 100 μl per well) or passive transfection (1 μM ODN, 100 μl per well) in reduced serum DMEM medium without antibiotics (above). After 24 h of lipid transfection or 72 h of passive transfection, cells were collected for luciferase assays. At this time, the medium was removed and the cells were washed with 1×D-PBS before completing a luciferase assay. The Promega Dual-Glo assay system (E2940, Madison, WI, USA) was used to measure firefly and renilla luciferase.

## Results

### Synthesis of peptide-substituted oligonucleotides

**Figure 1:**
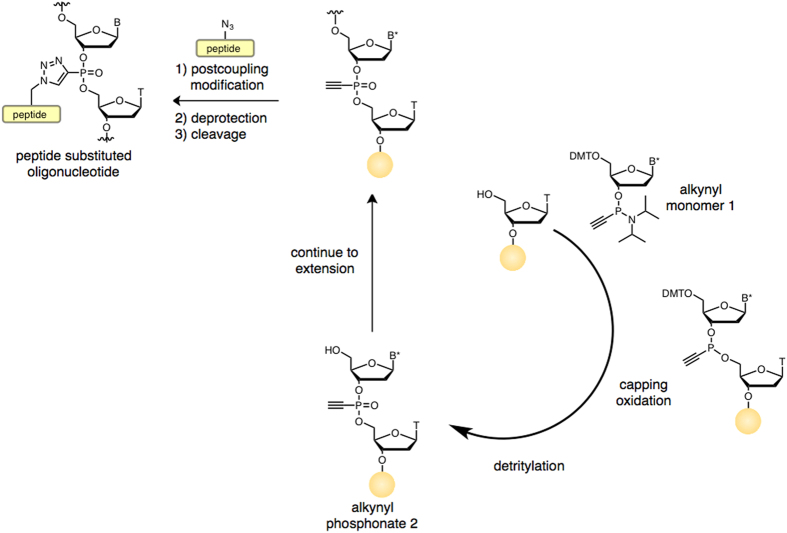
Synthetic strategy for TP ODNs having lysine or peptide-substituted 1,2,3-triazolylphosphonate internucleotide linkages. This solid-phase synthesis procedure introduces an alkynyl group at any desired position within the ODN backbone. B*=N^6^-benzoyladenine, N^4^-acetylcytosine, N^2^-isobutyroylguanine or thymine; B=adenine, cytosine, guanine or thymine.

The general synthetic strategy is outlined in Figure 1. Synthesis begins by condensing an alkynylphosphinoamidite 1 with a 2′-deoxynucleoside joined to an insoluble support. Following capping with acetic anhydride and iodine oxidation, the 5′-dimethoxytrityl group is removed and the cycle repeated. By incorporating either synthon 1 or a standard 2′-deoxynucleoside 3′-phosphoramidite, an oligonucleotide having both standard phosphate and alkynylphosphonate internucleotide linkages at any preselected position can be prepared. The final synthesis step is conversion of the alkynylphosphonates into a TP ODN analog. Thus, through click chemistry peptides having an azido group and an ODN bearing an alkynyl functionality can be joined together post-synthetically without protecting group manipulation. Final yields for the click chemistry coupling procedure exceed 85%. A summary of synthesized TP ODNs, their molecular weight analysis, and net charge/TP ODN is presented in Table 1.

Using the synthesis strategy as outlined in Figure 1 and presented in the experimental section, TP ODNs that were anti-miR oligonucleotides (see Table 3 and Figure 7) to miR-15b and having combinations of lysine or peptide triazolylphosphonate linkages as well as normal phosphate were synthesized. These TP ODNs were selected so that the modifications were located outside the seed region of miR-15b. This would allow perfect base pairing for microRNA recognition as part of the RNA-induced silencing complex (Figure 7a). The number of peptide/amino-acid substitutions ranged from two to six per TP ODN. In addition, each modification, due to peptide sequence variation, carried variable-positive charges at the pH used for cell culture experiments (7.4). For example, 3PJ would likely bear a near neutral (−3) net charge compared with the −21 charge expected from a natural phosphate backbone at pH 7.4. Various peptides were also enriched in lysine, arginine or histidine to initiate a study of the cell-penetrating assistance of TP ODNs.

### Thermal stability properties of TP ODNs

To verify the effect of TP ODNs on the stability of DNA duplexes, the *T*_m_s of these oligonucleotides were determined (Table 2). TP ODNs carrying a dipeptide modification provide better stability than a lysine modification (GR versus K). For TP ODNs carrying larger peptides, three modifications were less destabilizing than two (2PA versus 3PA, 2PG versus 3PG and 2PJ versus 3PJ). Moreover, among these peptide TP ODNs, lysine-rich TP ODNs (PA) showed less effect on hybridization when compared with arginine (PG)- and histidine (PJ)-rich TP ODNs. In addition, for certain peptide TP ODNs, (4GR) and (3PA), the Δ*T*_m_s were as low as −0.93 °C per modification and −0.80 °C per modification. Thus, these modifications exhibited results comparable to the known *T*_m_ reduction for thiophosphate-containing ODNs (−0.6 °C per modification).

### Flow cytometry of TP ODNs

**Figure 2:**
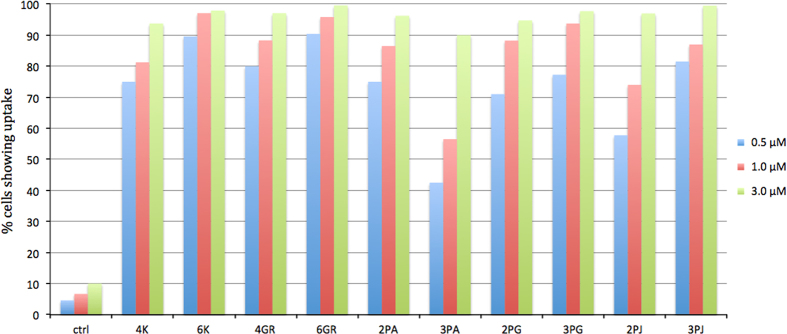
FL TP ODNs analyzed by FACS. Results are shown as the percentage of Hela cells with uptake. Control sequence is 5′-FL-TAGCAGCACATCATGGTTTACA-3′ with phosphate linkages. FL represents a fluorescein tag joined through a thiophosphate to the 5′-end of an oligonucleotide.

TP ODN transfection analysis was by fluorescence-activated cell sorting (FACS). HeLa cells and fresh medium were premixed with the TP ODNs at various concentrations (0.5, 1.0 and 3.0 μM) and the cells were incubated for 16 h. Fluorescence intensity was then compared with the autofluorescence of cells incubated with medium during the 16 h transfection time. Results are shown in Figure 2. In the absence of a lipid transfecting reagent, TP ODNs were taken up by HeLa cells in a dose-dependent manner. TP ODNs with smaller modifications (K and GR) were more efficiently taken up even at the lowest concentration tested (>70%). Although TP ODNs having larger peptide modifications (PA, PG and PJ) were less efficient, these ODNs still showed consistently higher uptake (>40%) than cells incubated with an unmodified control oligonucleotide.

### Passive and lipid-assisted transfection

**Figure 3:**
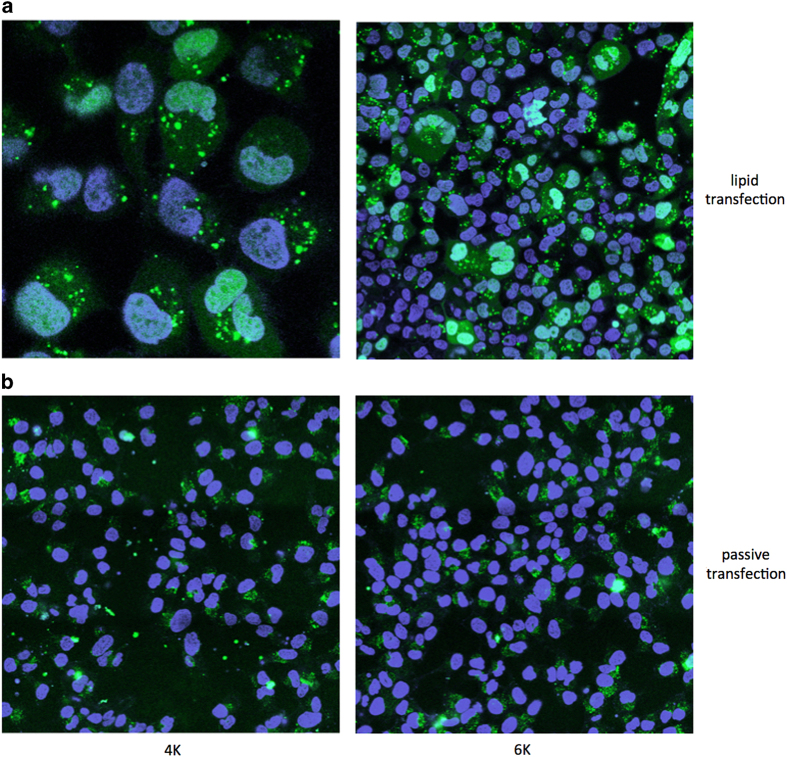
Localization of fluorescein-labeled ODNs in HeLa cells. (**a**) Lipid transfection: 100 nM fluorescein-labeled 4K (left) and 6K (right) were transfected into HeLa cells using DharmaFECT 1 (Dharmacon) reagent for 18 h followed by washing. (**b**) Passive transfection: HeLa cells were incubated with 1 μM fluorescein-labeled 4K (left) and 6K (right) for 90 h followed by washing. Cells were stained with Hoechst 33258 to visualize the nuclei.

To reveal the location of TP ODNs within cells and to exclude the possibility that these oligonucleotides were simply adhering to cell membranes, which would cause false positives by FACS analysis, HeLa cells were treated with fluorescein-labeled 4K and 6K TP ODNs in the presence or absence of a transfection reagent. Using fluorescence microscopy, these TP ODNs were localized in the nucleus and cytosol when delivered into the cells with cationic lipid (Figure 3a). In the absence of a lipid transfection reagent, there was no nuclear uptake; instead, the TP ODNs were distributed in a perinuclear region of cells (Figure 3b).

### Passively transfected ODNs localize near to a Golgi marker

**Figure 4:**
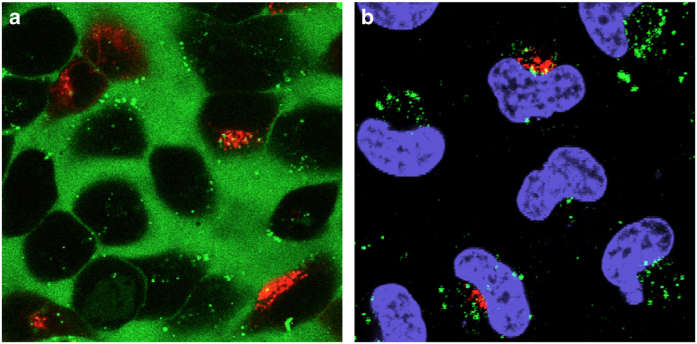
Localization of a fluorescein-labeled, unsubstituted TP ODN (5′-FL-T_u_A_u_ACACGATACGCG_u_A_u_T-3′, unsubstituted TP linkages are designated by u)^4^ with mCherry-Gal-T. After labeling HeLa cells with Gal-T using TransIT-LT1 for 30 min, cells were incubated with unsubstituted TP ODN at 37 °C and monitored continuously for 15 h. Cells were stained with Hoechst 33258 to visualize the nuclei. (**a**) Before washing ODN. (**b**) After washing ODN and nucleus staining. Confocal microscopic image merging showed the unsubstituted TP ODN localizes near to the Golgi marker.

Fluorescence microscopy showed that these passively transfected TP ODNs accumulate on one side of the nucleus and not throughout the entire cytoplasm. Further colocalization examination indicated that the TP ODNs distributed close to and likely interacting with the Golgi apparatus during 15 h of passive delivery (Figures 4a and b).

### Endosomal-releasing reagents improved cellular uptake

There are two cellular pathways that are responsible for oligonucleotide delivery in cells:^11^ a non-productive pathway resulting in the accumulation of oligonucleotide in intracellular compartments such as the Golgi and endosomes, and a functional pathway in which the oligonucleotides gain access to the cytosols and nucleus. Apparently, despite success in passive cell delivery, methods for efficient endosomal escape still remain a goal.

**Figure 5:**
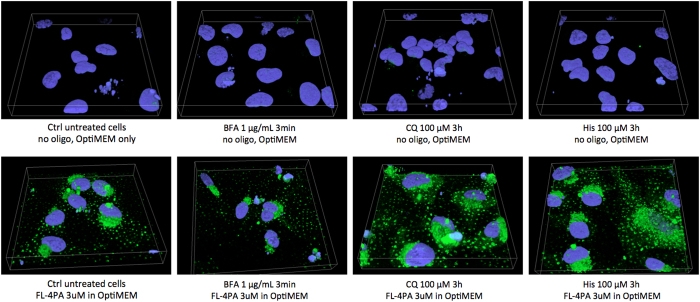
The effect of 1 μg ml^−1^ brefeldin A (BFA), 100 μM chloroquine (CQ) and 100 μM histidine (His) on endosomal release of a TP ODN (FL-4PA). Note that the suggested concentration of BFA is 10 μg ml^−1^ for endosomal release. In the research reported here, this concentration caused significant toxicity even after a short treatment. Thus, a lower and less toxic 1 μg ml^−1^ concentration was used for this experiment. Passive transfection was performed using 3 μM TP ODN FL-4PA for 24 h followed by washing. Cells were then incubated with BFA, CQ and His, respectively. Three-dimensional microscopy was taken at 0.5 μm per step for 30 steps. Fl-4PA: 5′-FL-T_PepA_GTAAAC_PepA_CATGA_PepA_TGTGCTGCT_PepA_A-3′. PepA, AcHN-Lys(N_3_)-Lys-Lys-Arg-Gly-NH_2_.

The Golgi apparatus is made of stacks of a membrane-bound structure whose function is to modify and package substances from the endoplasmic reticulum (ER), a complex series of tubules that is connected to the nuclear envelope. Therefore, the ER provides a crucial pipeline between the nucleus and the cytoplasm for exogenous oligonucleotide uptake. In attempts to release TP ODNs from these endosomal organelles, several pharmacological inhibitors were tested. These included CQ, an inhibitor of lysosome acidification; brefeldin A, a lactone antibiotic that redistributes proteins from the Golgi apparatus to the ER;^12^ and histidine, a non-toxic weak base that acts as an enhancer for better cellular uptake. Results using these drugs are shown in Figure 5. Although brefeldin A was effective in releasing entrapped TP ODNs, it was also toxic to the cells and not suitable for our prolonged passive transfections. Both CQ and histidine had similar releasing effects after 3 h of treatment but with low cytotoxicity (Figure 5).

### Cellular uptake is independent of cell division

**Figure 6:**
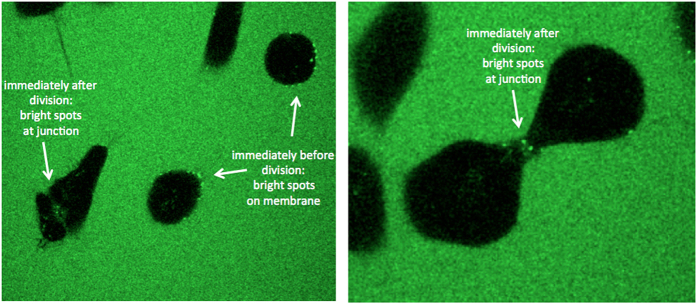
HeLa cells were arrested at the beginning of S phase using a double thymidine block. The synchronized cells then were incubated with 1 μM fluorescein-labeled 4PA and monitored continuously by confocal microscopy for 26 h. Pictures were taken every 10 min. Cell division started at ~11 h and peaked at 15–17 h.

To follow uptake kinetics of TP ODNs in real time, we have also prepared a movie (Supplementary Information) of synchronized cell behavior at division. We found that uptake of a TP ODN was not significantly improved during cell division, but bright TP ODN granules were observed at the cell membrane immediately before division, and at the splitting junction after division (Figure 6).

### Peptide-substituted TP ODNs can silence miRNA *in vitro*

**Figure 7:**
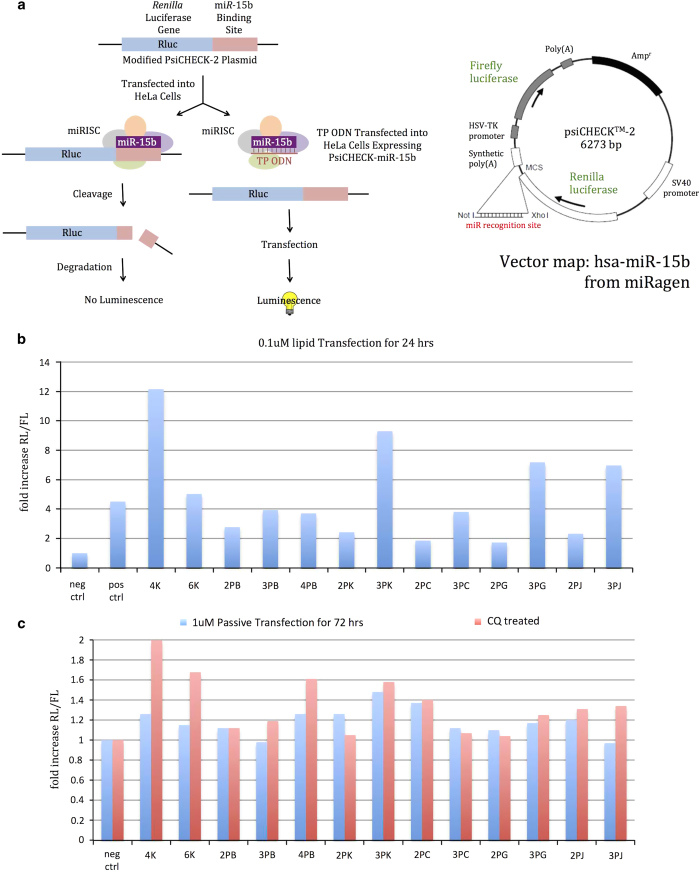
(**a**) A stably transfected vector in HeLa cells expresses two luciferase genes. Firefly luciferase is expressed constitutively, whereas expression of the renilla luciferase gene is under the control of miR-15b as part of the RNA-induced silencing complex (RISC). In the presence of an anti-miR-15b oligonucleotide (TP ODN in this figure), the RISC dissociates from the renilla gene. This leads to an increase in renilla luciferase activity. Comparison of the activity of renilla luciferase to the unregulated firefly luciferase yields an indication of the activity of an oligonucleotide as an anti-miR-15b oligonucleotide. (**a**) Adapted from the Integrated DNA Technologies Inc. (Coralville, IA, USA) website. The stably transfected HeLa cell line and assay protocols were developed by miRagen Therapeutics, Inc. (**b**) Lipid transfection using DharmaFECT 1 (Dharmacon). (**c**) Passive transfection using TP ODNs. Positive and negative controls were purchased from Dharmacon: miRIDIAN Hairpin Inhibitor (IH-300587-07) and miRIDIAN Hairpin Inhibitor Negative Control #1 (IN-001005-01).

To further probe the functional pathway involved in TP ODN delivery, the potencies of TP ODNs as anti-microRNA oligo-nucleotides to miR-15b (a potential therapeutic target for ischemic injury and myocardial infarcation^13^) were tested^14^ (Figure 7a). In the presence of a cationic lipid, certain single-stranded TP ODNs (for example, 4K, 3PK, 3PG and 3PJ) were very effective in silencing miR-15b as they generated higher activity than a commercially available, synthetic positive control (Figure 7b). Passively transfected cells showed a correlation with lipid-assisted transfection, especially after CQ treatment (Figure 7c). For example, many of the passively transfected TP ODNs were active in the presence of CQ (4PB, 3PK, 4K and 6K) with the highest (4K) being twofold more active than the negative control and about half as active as a commercially available, cholesterol-conjugated antagomir.^13^ Importantly, and unlike lipid-assisted transfection, none of the passively transfected TP ODNs in the presence of CQ showed significant cytotoxicity after 72 h. Although the 4K and 6K TP ODNs carry the highest net negative charge (Table 3), after entering cells they must escape in the presence of CQ and reach the target of interest. Conversely, 3PJ with a net negative charge of −3 did not yield significant bioactivity even with an impressive FACS uptake profile (Figure 2). Perhaps, bioavailability is a subtle balance between uptake and endosomal escape, which is precisely controlled by peptide sequence or net charge on a TP ODN. Because of similar compositions for the cell and endosomal compartment membranes, neutralized oligonucleotides may penetrate cells and endosomal compartments freely, but are more likely to be trapped. How to break this balance without damaging cells is the key to a successful therapeutic application or for using oligonucleotides to study the cell biology of cells *in vitro*.

## Discussion

A general problem associated with studying the efficacy of various modified oligonucleotides in cell culture relates to their uptake and activity. Generally, these oligonucleotides reside in intracellular, endosomal compartments where they are ineffective relative to displaying activity. This problem has led to the use of toxic lipids during transfection to facilitate the release of oligonucleotides into the cytoplasm where activity can be measured. However, lipid toxicity always limits an evaluation of the effectiveness of various oligonucleotides in controlling gene activity.

In this paper, we show that certain drugs, especially CQ when combined with TP ODNs having specific peptides or amino acids, facilitate the endosomal release of these ODNs into the cytoplasm. Of particular interest was the observation that lysine-rich peptides (4K, 6K and 4PB) show enhancement in activity. These observations are consistent with the known results that lysine-rich peptides are selectively trafficked to the ER and Golgi.^15^ Thus, it is possible that passive transfection of lysine-rich TP ODNs when coupled with release from Golgi/ER/endosomes by CQ could serve as a general method for introducing biologically active TP ODNs to cells in culture.

Interestingly, recent studies found that phosphorothioate oligonucleotides were primarily localized in GW/P bodies in a human melanoma cell line (Huh-7)^11^ or in lysosomes in a novel mouse hepatocellular SV40 large T-antigen carcinoma cell line (MHT).^16^ In the MHT cell line, researchers used CQ and brefeldin A as endocytosis inhibitors instead of endosomal-releasing reagents and observed increased cell uptake, but decreased functional uptake as measured by antisense effects to messenger RNA. However, it is difficult to compare the results with MHT cells to the data presented in this manuscript. Also, there is no evidence that phosphorthioate oligonucleotides and TP ODNs are being sequestered into the same cell organelles.

In conclusion, we have developed an efficient solid-phase synthesis strategy for preparing amino acid- and peptide-substituted TP ODNs that provide sequence and charge variations. The results demonstrate that these TP ODNs, via passive transfection, improve cellular uptake and silence miR-15b *in vitro*. This work addresses three important current challenges relative to ODN delivery. First, TP ODNs improve the physicochemical properties of natural phosphate DNAs by modifying the net negative charge and reducing sensitivity towards nuclease degradation (data not shown). Second, TP ODNs yield a library of oligonucleotide analogs that cross cell membranes without the help of carriers. Third, TP ODN delivery is highly accessible by cells as they generate significant activity via lipid transfection and a moderate increase in activity when passive transfection is combined with endosomal release using CQ. Expansion of this approach to provide a more comprehensive understanding and screening against a variety of cell types is ongoing.

## Figures and Tables

**Table 1:** TP ODNs: sequences, molecular weight analysis and net charge at pH 7.4

*TP ODN*	*Sequence*	*Molecular weight*		*Net charge*
		*Calculated*	*Observed*	
4K	5′-T_Lys_GTAA_Lys_ACC_Lys_ATGATGTGCTGCT_Lys_A-3′	7469.8	7468.3	−17
6K	5′-T_Lys_GTAAA_Lys_C_Lys_C_Lys_A_Lys_TGATGTGCTGCT_Lys_A-3′	7830.0	7828.9	−15
4GR	5′-T_GR_GTAA_GR_ACC_GR_ATGATGTGCTGCT_GR_A-3′	7806.0	7804.9	−13
6GR	5′-T_GR_GTAAA_GR_C_GR_C_GR_A_GR_TGATGTGCTGCT_GR_A-3′	8334.3	8333.8	−9
2PA	5′-T_PepA_GTAAACCATGATGTGCTGCT_PepA_A-3′	8130.3	8128.5	−13
3PA	5′ -T_PepA_GTAAACCA_PepA_TGATGTGCTGCT_PepA_A-3′	8820.6	8820.4	−9
2PG	5′-T_PepG_GTAAACCATGATGTGCTGCT_PepG_A-3′	8252.3	8251.7	−13
3PG	5′ -T_PepG_GTAAACCA_PepG_TGATGTGCTGCT_PepG_A-3′	8988.8	8988.4	−9
2PJ	5′-T_PepJ_GTAAACCATGATGTGCTGCT_PepJ_A-3′	8526.4	8524.9	−9
3PJ	5′-T_PepJ_GTAAACCA_PepJ_TGATGTGCTGCT_PepJ_A-3′	9414.9	9414.7	−3

Abbreviations: ACHN, acetylated N terminus; GR, N_3_-Gly-Arg-NH_2_; NH_2_, amidated C terminus; PepA, AcHN-Lys(N_3_)-Lys-Lys-Arg-Gly-NH_2_; PepG, AcHN-Lys(N_3_)-Arg-Arg-Arg-Gly-NH_2_; PepJ, AcHN-Lys(N_3_)-Lys-Lys-His-His-His-NH_2_; TP ODN, triazolylphosphonate oligodeoxynucleotide.

The MALDI measurements were obtained in the negative ion mode. The number preceding the peptide/amino-acid abbreviation refers to the number of peptide triazoylphosphonate internucleotide linkages/TP ODN.

**Table 2:** TP ODN melting temperatures

*ODN*	*Duplex*	*Modifications*	T*_m_*	*Δ*T*_m_*	*Δ*T*_m_ per mod*
ctrl	x′/x	0	70.0	—	—
4K	4K/x	4	63.0	−7.0	−1.75
6K	6K/x	6	56.6	−13.4	−2.23
4GR	4GR/x	4	66.3	−3.7	−0.93
6GR	6GR/x	6	59.0	−11.0	−1.83
2PA	2PA/x	2	66.6	−3.4	−1.70
3PA	3PA/x	3	67.6	−2.4	−0.80
2PG	2PG/x	2	54.6	−15.4	−7.70
3PG	3PG/x	3	64.3	−5.7	−1.90
2PJ	2PJ/x	2	61.0	−9.0	−4.50
3PJ	3PJ/x	3	62.0	−8.0	−2.67

Abbreviations: mod, modification; TP ODN, triazolylphosphonate oligodeoxynucleotide; x, 5′-TAGCAGCACATCATGGTTTACA-3′; x′, 5′-TGTAAACCATGATGTGCTGCTA-3′.

The TP ODN sequences are defined in Table 1. All *T*_m_s represent an average of at least three experiments; Δ*T*_m_ is the difference in *T*_m_ when compared with *T*_m_ of the unmodified duplex.

**Table 3:** Sequences of the TP ODNs studied in the luciferase assay

*Oligo*	*Sequence*	*Net charge*
4K	5′-T_Lys_GTAA_Lys_ACC_Lys_ATGATGTGCTGCT_Lys_A-3′	−17
6K	5′-T_Lys_GTAAA_Lys_C_Lys_C_Lys_A_Lys_TGATGTGCTGCT_Lys_A-3′	−15
2PB	5′-T_PepB_GTAAACCATGATGTGCTGCT_PepB_A-3′	−13
3PB	5′ -T_PepB_GTAAAC_PepB_CATGATGTGCTGCT_PepB_A-3′	−9
4PB	5′-T_PepB_GTA_PepB_AACC_PepB_ATGATGTGCTGCT_PepB_A-3′	−5
2PK	5′-T_PepK_GTAAACCATGATGTGCTGCT_PepK_A-3′	−13
3PK	5′-T_PepK_GTAAAC_PepK_CATGATGTGCTGCT_PepK_A-3′	−9
2PC	5′-T_PepC_GTAAACCATGATGTGCTGCT_PepC_A-3′	−11
3PC	5′ -T_PepC_GTAAACCA_PepC_TGATGTGCTGCT_PepC_A-3′	−6
2PG	5′-T_PepG_GTAAACCATGATGTGCTGCT_PepG_A-3′	−13
3PG	5′ -T_PepG_GTAAACCA_PepG_TGATGTGCTGCT_PepG_A-3′	−9
2PJ	5′-T_PepJ_GTAAACCATGATGTGCTGCT_PepJ_A-3′	−9
3PJ	5′-T_PepJ_GTAAACCA_PepJ_TGATGTGCTGCT_PepJ_A-3′	−3

Abbreviations: ACHN, acetylated N terminus; NH_2_, amidated C terminus; PepB, AcHN-Lys-Lys-Lys(N_3_)-Lys-Gly-NH_2_; PepK, N_3_-Gly-Arg-Arg-Arg-Gly-NH_2_; PepC, AcHN-Gly-Lys(N_3_)-Gly-Arg-Arg-Gly-Arg-Arg-Gly-NH_2_; PepG, AcHN-Lys(N_3_)-Arg-Arg-Arg-Gly-NH_2_; PepJ, AcHN-Lys(N_3_)-Lys-Lys-His-His-His-NH_2_; TP ODN, triazolylphosphonate oligodeoxynucleotide.

These sequences are anti-miR oligonucleotides (AMOs) to miR-15b (see Figure 7).
